# Chemical characterization and effect of *Ziziphora clinopodioides* green‐synthesized silver nanoparticles on cytotoxicity, antioxidant, and antidiabetic activities in streptozotocin‐induced hepatotoxicity in Wistar diabetic male rats

**DOI:** 10.1002/fsn3.4008

**Published:** 2024-03-28

**Authors:** Elham Pirabbasi, Mohammad Mahdi Zangeneh, Akram Zangeneh, Rohallah Moradi, Mojtaba Kalantar

**Affiliations:** ^1^ Department of Nutrition Shoushtar Faculty of Medical Sciences Shoushtar Iran; ^2^ Biotechnology and Medicinal Plants Research Center Ilam University of Medical Sciences Ilam Iran; ^3^ Shoushtar Faculty of Medical Sciences Shoushtar Iran

**Keywords:** diabetes, liver, silver, Streptozotocin, *Ziziphora clinopodioides* extract

## Abstract

The present research studied the cytotoxicity, antioxidant, and antidiabetic activities of biogenically synthesized silver nanoparticles (AgNPs) using *Ziziphora clinopodioides* (*Z. clinopodioides*) as a green mediator. Scanning electron microscopy (SEM), transmission electron microscopy (TEM), and ultraviolet–visible spectroscopy (UV–Vis) were employed to determine AgNPs. In the in vivo experiment, the model rats were categorized into different groups receiving 50, 100, 200, and 400 μg/kg of AgNPs and diabetic, positive, and normal groups (*n* = 10) using a random division. A single dose of streptozotocin (STZ) at 60 mg/kg was administered to induce diabetes and hepatotoxicity in rats. The administration of AgNPs was performed via intragastric administration for 25 days. On the final day, the levels of glucose and biochemical enzymes, namely aspartate aminotransferase (AST), alkaline phosphatase (ALP), alanine transaminase (ALT), and gamma‐glutamyltransferase (GGT), were assessed in the serum. Following tissue processing, liver sections with a thickness of 5 μm were prepared. Later, the total volume of different liver components, such as hepatocytes, sinusoids, portal vein, central vein, hepatic arteries, and bile ducts, was measured. The portal vein and bile duct volumes did not vary significantly in groups treated by AgNPs. However, the volume of the central vein and hepatic arteries exhibited noticeable variations in groups treated by AgNPs. After administration of streptozotocin, the volume of hepatocytes and sinusoids increased significantly. However, treatment with a high dose of AgNPs significantly decreased the volume of hepatocytes and sinusoids. In diabetic rats, administering AgNPs reduced the fasting blood glucose levels compared to the model group. In addition, AgNPs decreased the elevated levels of AST and ALP enzymes in a manner that depended on the dosage of AgNPs used. This research demonstrates the hepatoprotective and antidiabetic properties of AgNPs, suggesting their potential implications as hepatoprotective and antidiabetic supplements to prevent diabetes.

## Graphical abstract

It is recommended to use *Ziziphora clinopodioides* extract because of the hepatoprotective and antidiabetic properties of the AgNPs.

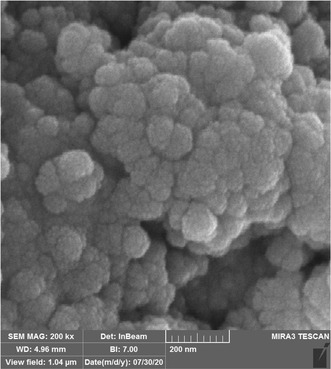

## 1 INTRODUCTION

Nanotechnology is an emerging scientific area with significant implications in academia and industry. Various physical, chemical, and biological methods have been employed to synthesize nanoparticles. However, physical methods are expensive and often involve high pressure and temperature (Balint et al., 2014; Balogh et al., 2007; Bardhan et al., 2011; Bartneck et al., 2012), while chemical methods can be harmful to the environment and biological systems. To address these challenges, the adoption of cost‐effective and environmentally friendly synthesis pathways is crucial (Annadhasan et al., 2015; Balasubramanian et al., 2010). Biological methods utilizing different microorganisms, plants, and plant extracts have gained attention due to their safety and simplicity (Almeida et al., 2014; Alric et al., 2013; Aminabad et al., 2019). Among these, the use of plant extracts offers advantages such as using water solvents, low environmental impact (Ahmad et al., 2016; Almeida et al., 2011; Connor et al., 2005), and compatibility with an abundance of biological materials (Ahmad et al., 2016; Almeida et al., 2011, 2014). Factors such as the properties of biological substances, duration of reaction, thermal conditions, acidity or alkalinity, concentration of metal salts, and concentration of extracts influence the production and characteristics of nanoparticles (Ahmad et al., 2016; Connor et al., 2005; Hanato et al., 2009; Li et al., 2018).

Nanoparticles have also found extensive implications in medicine, such as preventing, treating, and diagnosing diseases, using medical sensors, imaging, and employing drug delivery systems (Balogh et al., 2007; Bardhan et al., 2011; Bartneck et al., 2012). In particular, nanocomposites have shown anti‐diabetic properties and potential for delivering drugs to treat diabetes (Aminabad et al., 2019; Annadhasan et al., 2015; Balasubramanian et al., 2010; Balint et al., 2014). The encapsulation of diabetic drugs in nanoparticles enhances their efficiency in diabetic cells while reducing toxicity and side effects on healthy cells (Ahmad et al., 2016; Almeida et al., 2011, 2014; Alric et al., 2013). Targeted delivery of anti‐diabetic drugs to diabetic tissues with minimal impact on normal cells is crucial (Connor et al., 2005; Li et al., 2018). To this end, new delivery methods have been developed to specifically target drug delivery to diabetic tissues and reduce their side effects using nanoparticles as carriers (Ahmad et al., 2016; Araújo et al., 2014; Hanato et al., 2009; Kaasalainen et al., 2015). Nanoparticles from 10 to 100 nm were widely used for targeted destruction of diabetic cells, reducing systemic toxicity (Araújo et al., 2016; Hamelian et al., 2018; Hemmati et al., 2019; Hilger & Kaiser, 2012). Silver nanoparticles (AgNPs) are a commonly used photocatalyst with proven biocompatibility, resistance to corrosion (Cui et al., 2013; Hou et al., 2015; Idris et al., 2014; Lucky et al., 2015), and economic viability (Pérez‐Ortiz et al., 2017; Seo et al., 2007). AgNPs exhibit oxidation‐regeneration ability and can modify surface properties to a hydrophilic state (Nurunnabi et al., 2017; Oh et al., 2003; Rothman et al., 2005). They are attractive for delivering small molecules and biomolecules (Tamoradi et al., 2020; Veisi, Tamoradi, et al., 2019; Veisi et al., 2020). AgNPs can produce reactive oxygen species upon exposure to light, which can damage membrane lipids (Nasir Baig & Varma, 2013; Tamoradi et al., 2017; Veisi et al., 2018; Veisi, Ghorbani, et al., 2019). In this study, we synthesized AgNPs from *Z*. *clinopodioides* extract as an eco‐friendly agent for their reducing and stabilizing impacts. The synthesized AgNPs demonstrated potential preventive effects on streptozotocin‐induced diabetes and hepatotoxicity in rats.

## 2 MATERIALS AND METHODS

### 2.1 Preparation of *Z. clinopodioides* extract

After harvesting and drying one kilogram of *Z. clinopodioides* leaves, approximately 100 grams of dried leaves were obtained. Later, the dried leaves were ground, and extraction was performed after mixing the plant powder (15 gr) with distilled water (200 mL) in an Erlenmeyer flask. The resulting mixture was left to soak for three days. Afterward, the solution was strained using a strainer, and the solvent was evaporated in a water bath at a temperature of 40–50°C using wide plates. To make a solution of Dulbecco's Modified Eagle Medium (DMEM) at a concentration of 10 mg/mL, the extra solid material was employed (Hemmati et al., 2019).

### 2.2 Synthesis of the AgNPs

For synthesizing the AgNPs, a solution consisting of 50 g of the aqueous extract and 100 mL of AgNO_3_ × H_2_O (1 mM) solution was prepared. The solutions were then reacted at 80°C and 3000 rpm for 30 min. After 24 h, the reaction solution was exposed to centrifugation at a speed of 10,000 rpm for a duration of 15 min, and the resulting sediment was rinsed with deionized water. In order to make sure about the complete removal of contaminants, this washing process was repeated three times. Subsequently, the sediment was transferred to an oven set at a temperature of 60°C and left to dry for 24 h.

Field Emission Scanning Electron Microscopy (FE‐SEM) was recruited to confirm the crystalline and spherical characteristics of the silver nanoparticles (Hemmati et al., 2019).

### 2.3 2,2‐Diphenyl‐1‐picrylhydrazyl (DPPH) assay protocol

As a widely utilized technique, the DPPH assay assesses the antioxidant activity of different compounds. This assay works by changing the color spectrum of the DPPH free radicals from purple to yellow upon hydrogen absorption, reducing the absorption rate at the wavelength of 517 nm. To make the working solution ready, DPPH powder (2 mg) was dissolved in ethanol (30 mL). By utilizing serial dilution, various concentrations of nanoparticles were combined with an equal volume of the DPPH free radical in microtubes to assess the inhibition of free radicals by the nanoparticles. Ethanol served as the negative control, while butylated hydroxytoluene (BHT), a synthetic antioxidant, served as the positive control. The IC50 index, which represents the concentration of a substance required to achieve a 50% inhibition of free radicals, was calculated to evaluate the free radical inhibition (Hamelian et al., 2018; Hemmati et al., 2019).

$$\text{Inhibition}\;\left(\%\right)=\frac{\text{Sample}\;A.}{\text{Control}\;A.\;}\times 100$$

### 2.4 [3‐(4,5‐Dimethylthiazol‐2‐yl)‐2,5‐diphenyl tetrazolium bromide] (MTT) assay protocol

The normal cell line employed in the present investigation was human umbilical vein endothelial cells (HUVEC), purchased from the Pasteur Institute of Iran (IPI). The HUVEC cells were cultured in 1640‐RPMI medium from GiBco, supplemented with bovine serum (10%), streptomycin and penicillin antibiotics (1%), and glutamine (2%). The cell culture flasks were maintained in an incubator set at a temperature of 37°C with 5% CO_2_ and 95% humidity. The culture medium was refreshed every three days, and the flasks were maintained at an approximate cell density of 80% (cells covering 80% of the bottom surface) for the experiments. To detach the cells from the flask, the culture medium was removed, and a volume of 1 mL of trypsin was introduced and left for a duration of 3 min. Later, an equivalent amount of medium was included to counteract the effects of the trypsin. The cell suspension was subjected to centrifugation at a speed of 1200 revolutions per minute (rpm) for 4 min, and the supplement was discarded. The sediment was resuspended in 1 mL of culture medium. To determine the number of viable cells, a volume of 10 μL from the cell suspension was combined with an equal amount of trypan blue on a Neobar slide, and the living cells were counted. For instance, each well of a 96‐well plate received 10,000 cells from the cell suspension, followed by the addition of 180 μL of culture medium to each well. The concentration of nanoparticles used was within the range of 0–1000 μg/mL. A control group without nanoparticles, where water was added instead, was also included. Each experiment was performed in four replicates.

Following 24, 48, and 72 h of incubation, the existing medium in the wells was substituted with fresh medium. Later, 20 μL of MTT solution was introduced into each well and incubated. The reaction of the viable cells' mitochondrial succinate dehydrogenase enzyme with MTT solution led to the formation of insoluble purple formazan crystals.

Subsequently, we added 200 μL of dimethylsulfoxide (DMSO) to each well to dissolve the formazan crystals and shook the plate for 20 min to make sure about its thorough dissolution. An enzyme‐linked immunosorbent assay (ELISA) reader was employed to measure the solution's absorbance at wavelengths of 492 nm and 630 nm. At the end, the cell viability percentage was assessed by dividing the treated cells' optical absorbance (OD) by the control cells' OD and multiplying the result by 100 (Hamelian et al., 2018; Hemmati et al., 2019).

### 2.5 In vivo design

Diabetes was induced in 90 rats, weighted at about 210 ± 5 g, using 60 mg/kg of STZ in 70 rats. Diabetes was defined as a blood glucose level higher than 250 mg/dL. Later, these rats were classified into seven subgroups, consisting of healthy (negative) rats that received distilled water, untreated negative control rats that received distilled water, positive control rats that received 60 mg/kg of Glibenclamide, and four groups of rats receiving nanoparticles at concentrations of 50, 100, 200, and 400 μg/kg (Zangeneh et al., 2018). After 20 days of treatment, the biochemical and stereological parameters of the rats were investigated using their blood samples (Zangeneh et al., 2018).

The stereological procedure of the study involved the dissection and rinsing of the rats' livers collected from each group (*n* = 5) using a cold saline solution to eliminate any trace of blood. The livers were weighed and placed in a solution of 10% neutral buffer to undergo the process of fixation. To obtain the liver volume, the liver samples were dipped into the water for 72 h. In the following, the orientator method was employed to cut 7–10 slabs from each liver sample.

After embedding these slabs into paraffin and preparing sections with 5 μm thickness, hematoxylin and eosin were used to stain these sections. The estimation of volume density for various liver structures, including hepatocytes, sinusoids, central veins, portal veins, hepatic arteries, and bile ducts, was conducted using a point‐counting method. For this estimation, microscopic images taken from one section of each liver were represented on 15 × 15 cm point probes. This projection was accomplished using a video projector connected to a microscope with an attached camera (Dinocapture ver.5, dino‐lit.com 30.5 mm) set at a total magnification of 2000×, which was used to count the points that intersected the desired structures within the field of view. The volume density was then estimated by the following formula:

$$\mathrm{Vv}=P_{\text{structure}}/P_{\text{reference}}$$

where *Vv* represents the volume density of the liver structure, and *P*_structure_ and *P*_reference_ are the numbers of points falling on the structure's profile and on the reference space, respectively.

This method allows for the estimation of the relative volume occupied by each specific liver structure. It provided quantitative information about the proportion of different liver structures (10–14 microscopic fields were examined for each liver) within the liver tissue. To avoid the reference trap, the absolute value of the liver structures was determined by multiplying the fractional volume with the final volume of the liver (Zangeneh et al., 2018).

In order to perform data analysis, one‐way analysis of variance and Duncan post hoc tests were run via SPSS‐22 software (*p* ≤ .05).

## 3 RESULTS AND DISCUSSION

**FIGURE 1:**
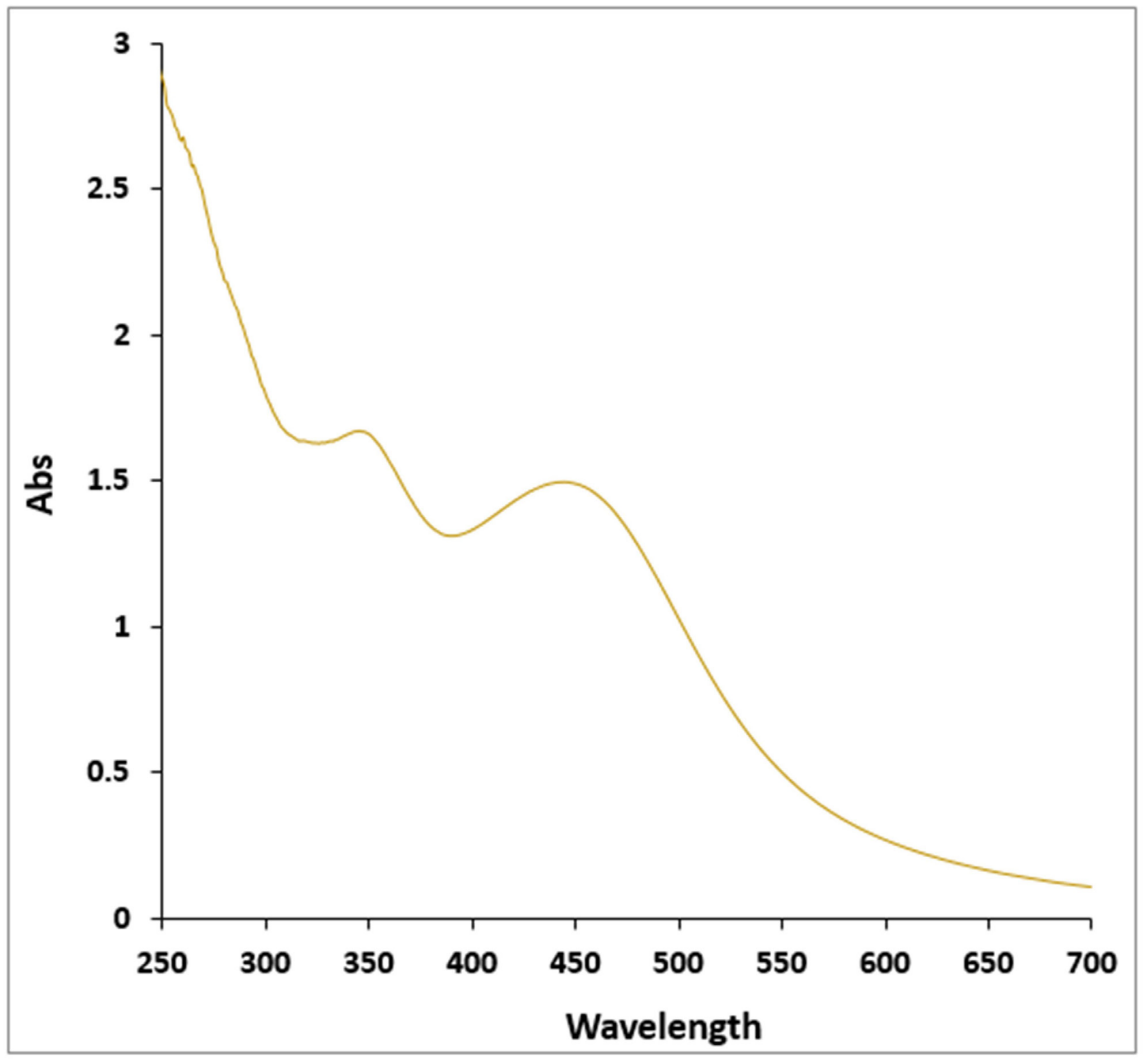
The UV–Vis pattern of Ag nanoparticles.

The provided information describes the results of an experimental study on the effects of AgNPs in a diabetic rat model. Based on the results, AgNPs were successfully synthesized and confirmed by the UV–Vis spectrum, showing a characteristic band at 441 nm (Figure 1).

The FE‐SEM analysis, as a widely applied scanning electron microscope, performs different samples' surface analysis and characterization. In FE‐SEM, a focused beam of electrons is scanned across the sample surface, and the interaction between the electrons and the sample generates signals that are used to create an image. The image quality and resolution obtained in FE‐SEM analysis are influenced by the samples' structure, synthesis quality, and absence of contamination or unwanted particles. Samples with well‐defined structures and minimal contamination tend to provide clearer and more detailed images. The electron beam's specific energy and wavelength also play a role in determining the image quality (Hamelian et al., 2018).

**FIGURE 2:**
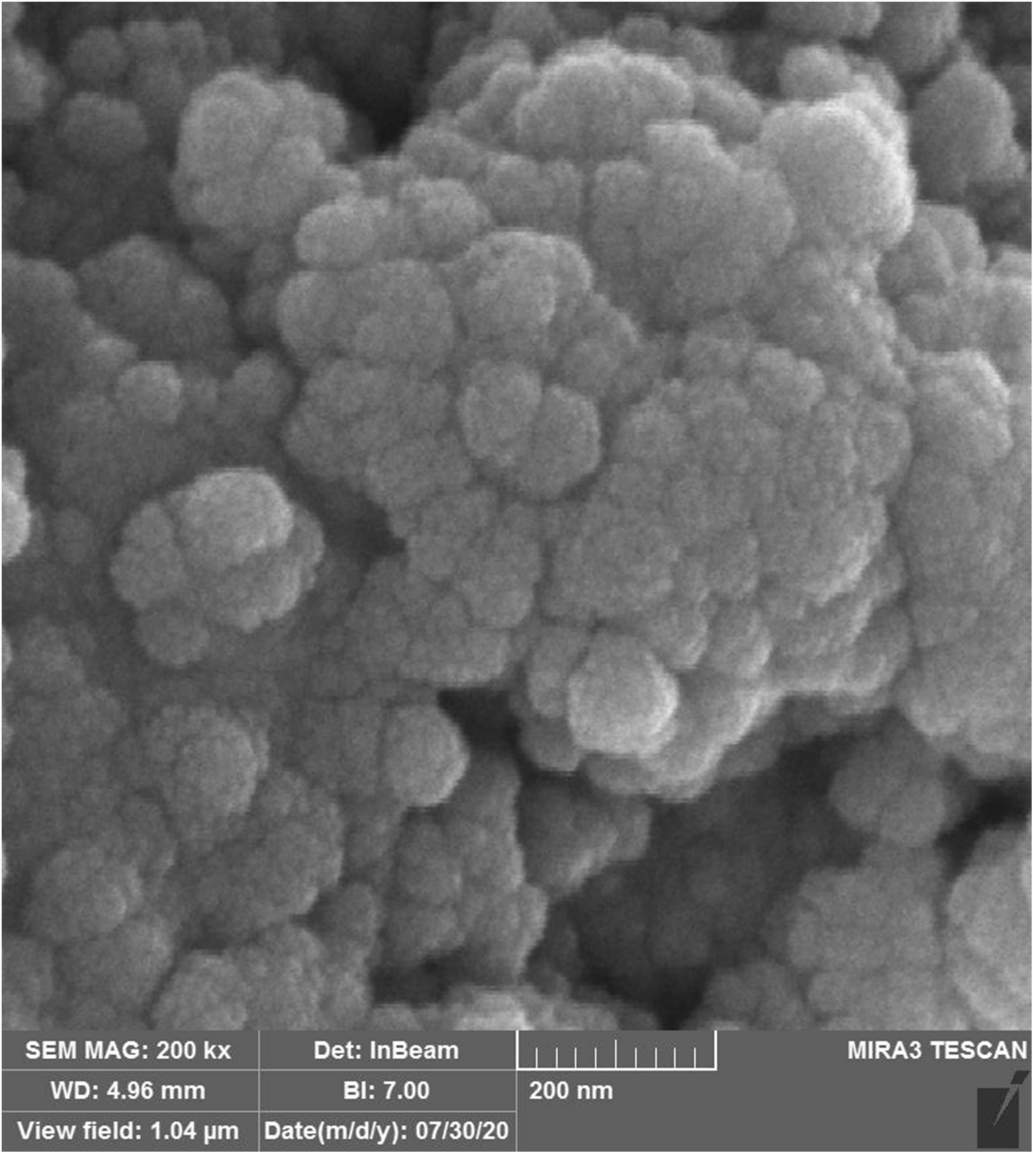
The FE‐SEM image of Ag nanoparticles.

**FIGURE 3:**
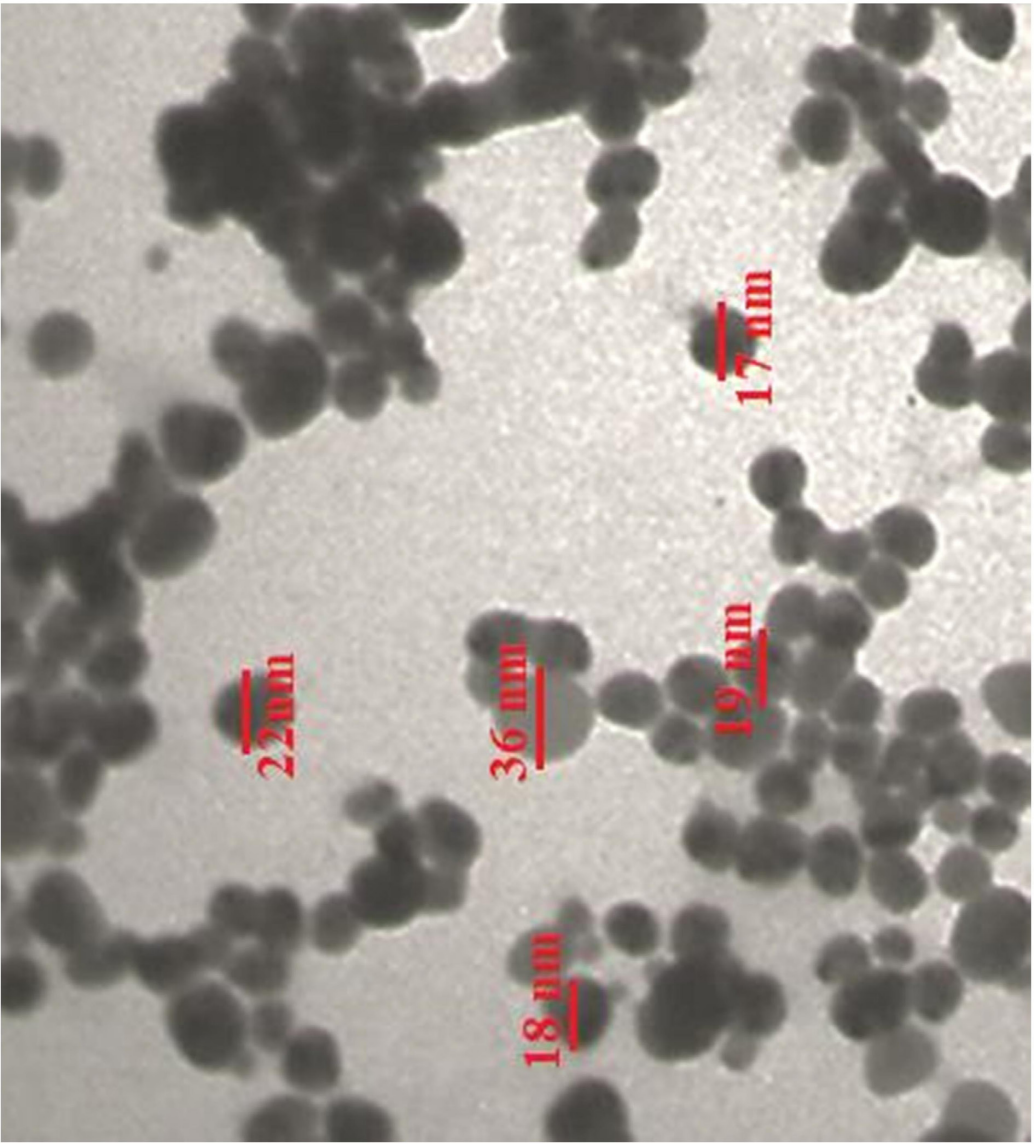
The TEM image of Ag nanoparticles.

FE‐SEM and TEM images of the AgNPs (Figures 2 and 3) revealed spherical nanoparticles with sizes ranging below 100 nm. Aggregation of the nanoparticles was observed, which is a common characteristic of metallic nanoparticles (Zangeneh et al., 2019). Previous studies have also reported that the size of AgNPs ranged from 10 to 100 nm (Hamelian et al., 2020).

**FIGURE 4:**
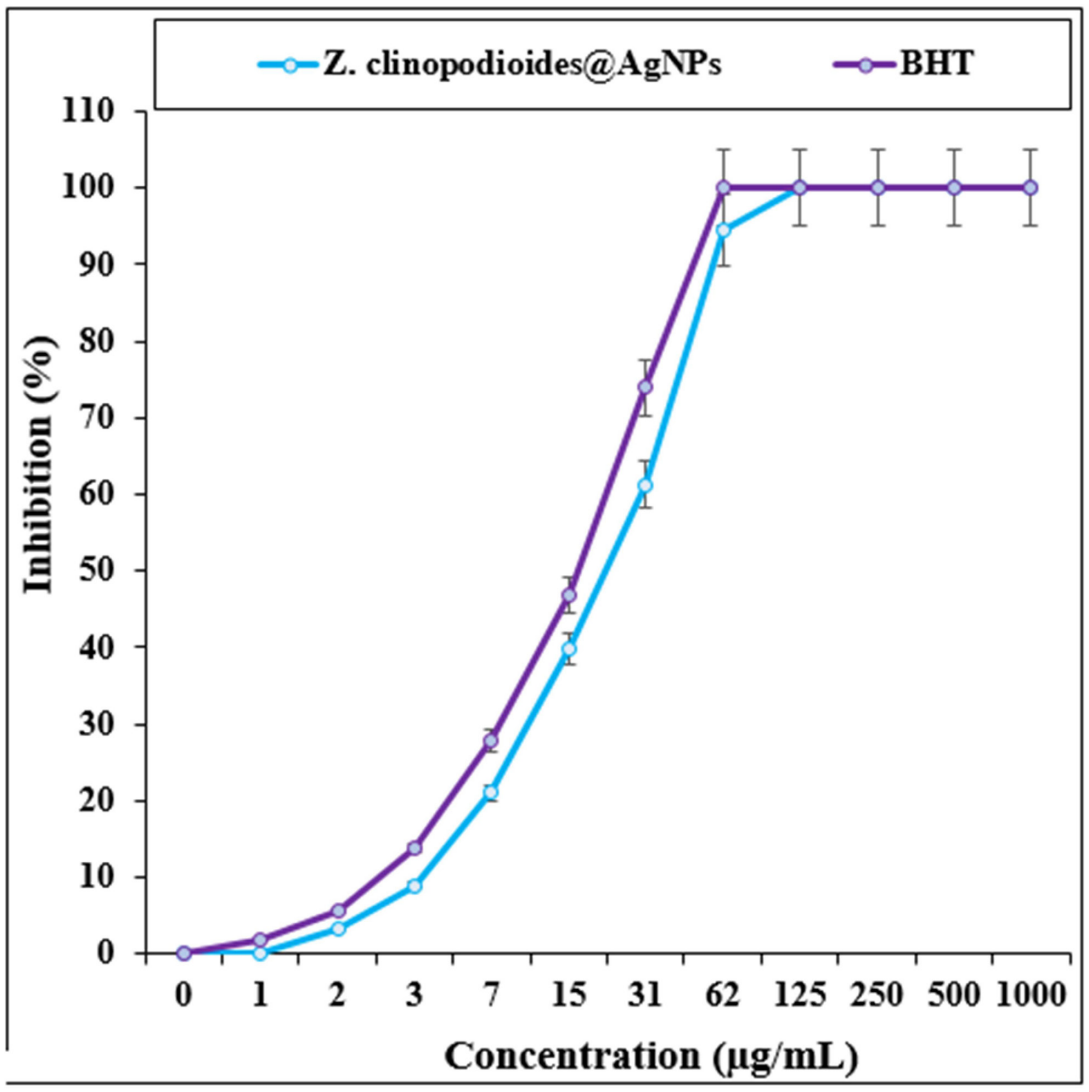
The antioxidant properties of *Ziziphora clinopodioides @*AgNPs and BHT against DPPH.

The scavenging capacity of AgNPs and BHT as an antioxidant was assessed using DPPH free radicals. The IC50 values of AgNPs and BHT were determined to be 22 μg/mL and 8 μg/mL, respectively (Figure 4).

**FIGURE 5:**
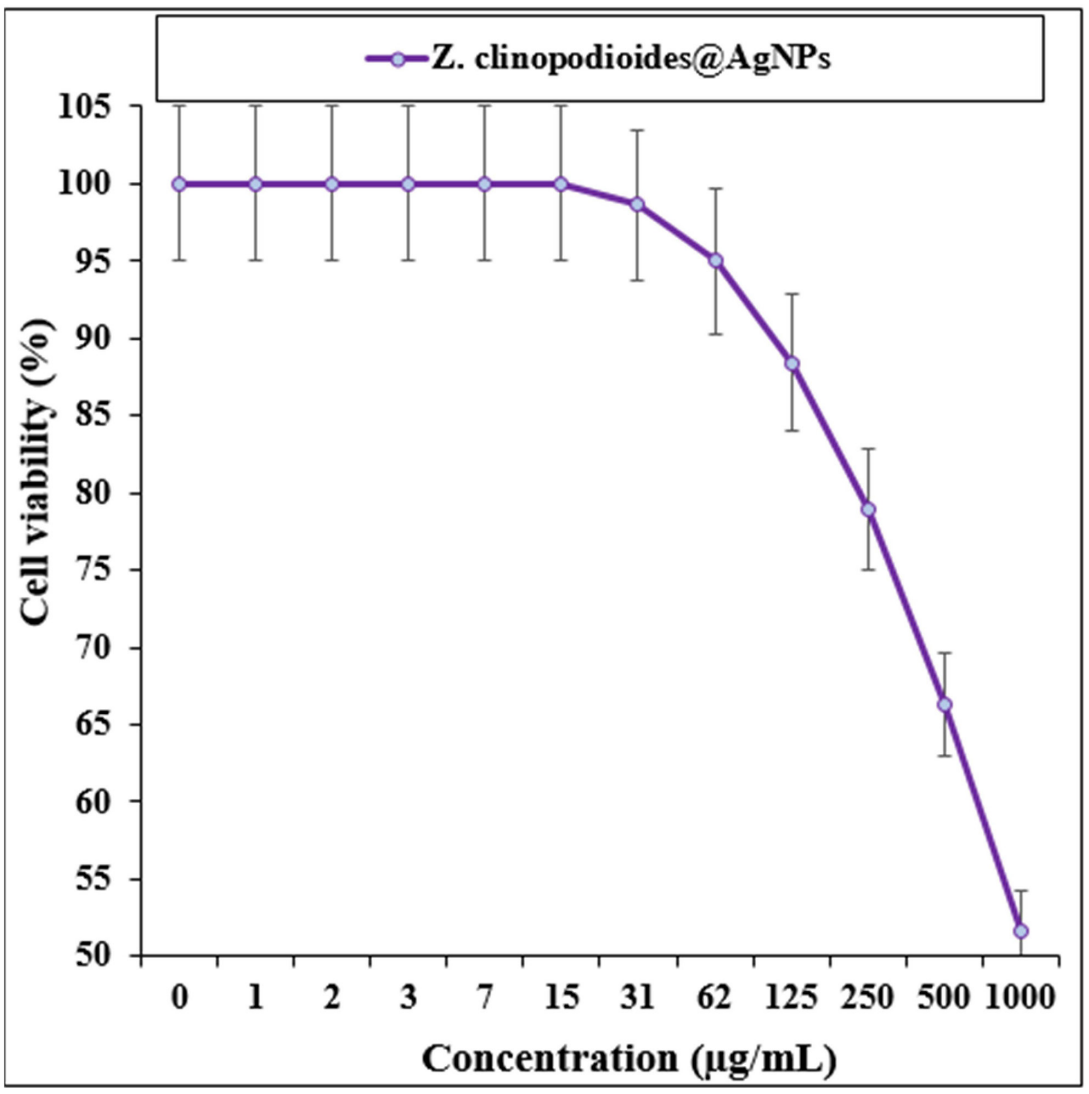
The cytotoxicity activities of *Ziziphora clinopodioides* and AgNPs against the HUVEC (normal) cell line.

The cytotoxicity test on HUVEC showed that AgNPs were not effective on cell viability even at a concentration of 1000 μg/mL (Figure 5).

From a theoretical point of view, nanomaterials, including nanoparticles, can be more toxic than microcompounds because of their great surface activity and potential for cellular penetration and accumulation (Balint et al., 2014; Balogh et al., 2007; Bardhan et al., 2011; Bartneck et al., 2012). In this regard, oxidative stress is critically important in inducing the cytotoxicity of nanoparticles in human cancer cells (Balasubramanian et al., 2010; Balint et al., 2014; Balogh et al., 2007). Medicinal plants possessing anti‐inflammatory and antioxidant properties, combined with the production of metal nanomaterials using plant‐active compounds, have been explored as a new approach to enhance their therapeutic effects and minimize side effects (Almeida et al., 2011, 2014; Alric et al., 2013; Aminabad et al., 2019; Annadhasan et al., 2015). For example, administration of a high concentration of nanoparticles over several months can result in anemia, pancreatic failure, and a decrease in dense lipids in the body (Aminabad et al., 2019; Annadhasan et al., 2015; Balasubramanian et al., 2010). However, a histopathological study on the liver and pancreas showed that the concentration of nanoparticles synthesized through biological methods, despite having anti‐diabetic properties, did not cause any significant toxic effects on the liver and pancreas tissues (Almeida et al., 2014; Alric et al., 2013; Aminabad et al., 2019; Annadhasan et al., 2015).

Various studies have revealed the protective impacts of medicinal plants on different human tissues, such as the liver, pancreas, and kidney. These plants can help protect and support the healthy functioning of human organs since they contain bioactive compounds, such as flavonoid, anthocyanin, and phenolic compounds with antioxidant and anti‐inflammatory properties (Ahmad et al., 2016; Connor et al., 2005; Hanato et al., 2009; Li et al., 2018). In examining the impact of various nanoparticles on the body, nanoparticles, considering their small size and unique properties, have the potential for easy penetration into cells and tissues (Balint et al., 2014, Balogh et al., 2007, Bardhan et al., 2011, Bartneck et al., 2012). While some scholars have reported potential adverse effects of nanoparticles, including serious damage, the toxicity and potential harms associated with nanoparticles can be justified by the specific nanoparticle material, the route of exposure, the duration of exposure, and the target tissue or organ (Alric et al., 2013; Aminabad et al., 2019; Annadhasan et al., 2015; Balasubramanian et al., 2010; Balint et al., 2014).

**FIGURE 6:**
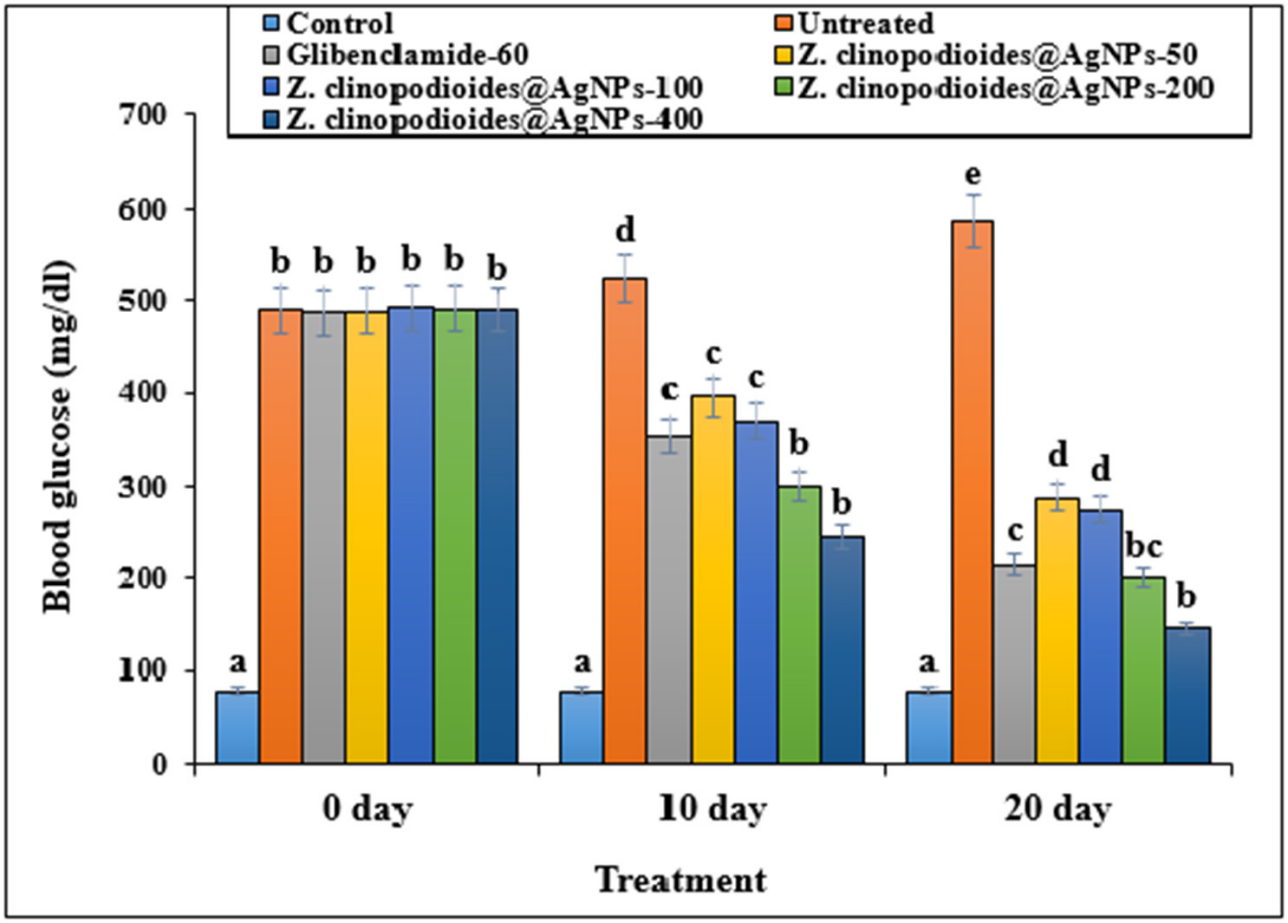
Blood glucose levels on 0, 10, and 20 days in several groups. The non‐similar letters indicate the significant difference between several groups (*p* ≤ .05).

As illustrated in Figure 6, the intake of AgNPs could increase blood glucose levels in the diabetic rats by almost 600% (*p* ≤ .05) time‐dependently. However, administrating all doses of AgNPs significantly reduced the FBS levels in STZ‐diabetic rats (*p* ≤ .05). The same results were achieved by the glibenclamide‐treated models. Intake of different AgNP doses had significant results (*p* ≤ .05) at 10 and 20 days, so the best effects were observed on day 20 of the experiment.

**FIGURE 7:**
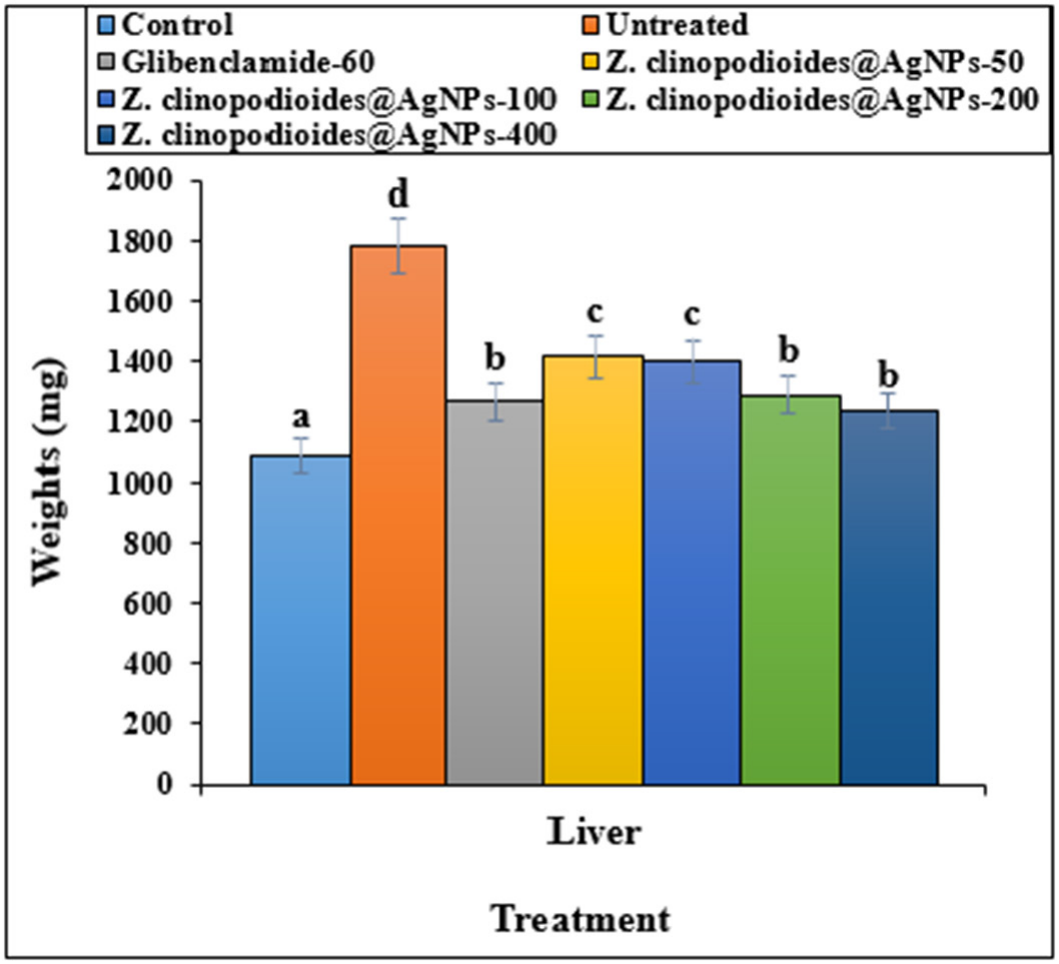
Liver weight levels in several groups. The non‐similar letters indicate the significant difference between several groups (*p* ≤ .05).

**FIGURE 8:**
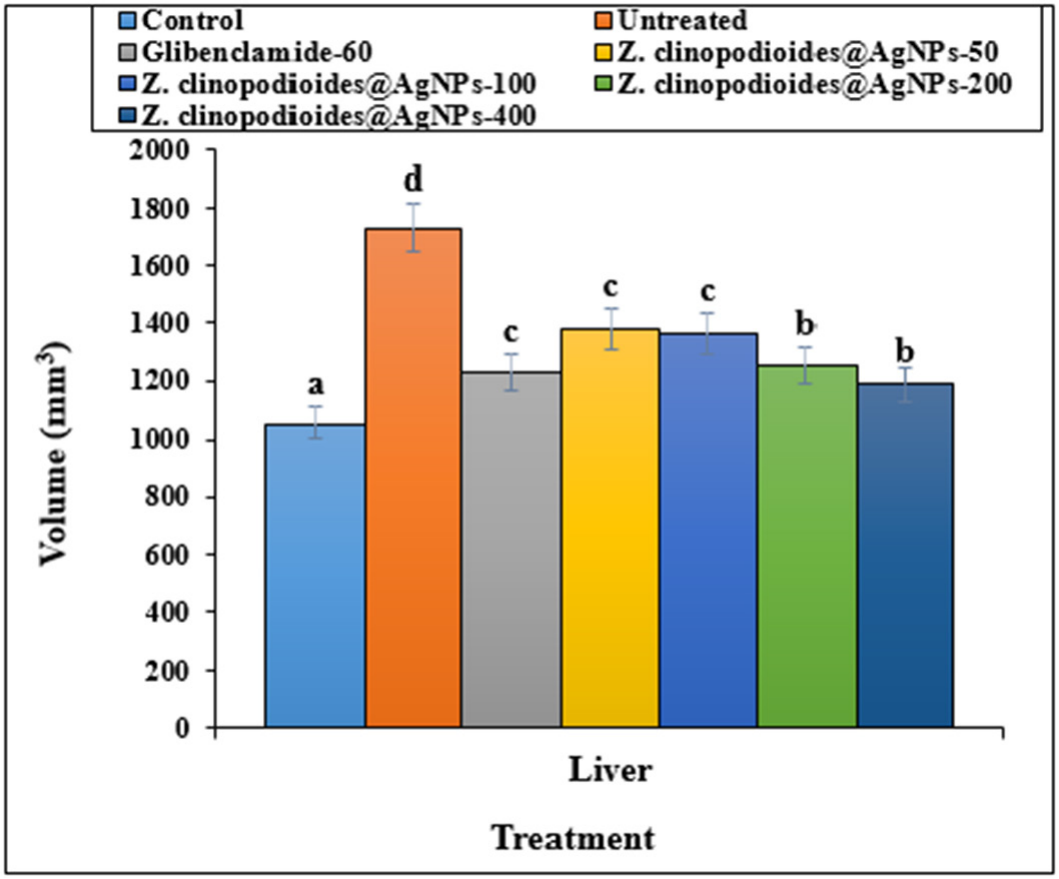
Volumes of liver in several groups. The non‐similar letters indicate the significant difference between several groups (*p* ≤ .05).

Figures 7 and 8 represent the mean absolute weights and volumes of the untreated and treated models' livers. Based on these results, administering all doses of AgNPs improved the liver volume and weight significantly (*p* ≤ .05); no significant difference was seen (*p* ≤ .05) in administration of AgNPs‐200, AgNPs‐400, and glibenclamide in the liver volume and weight.

**FIGURE 9:**
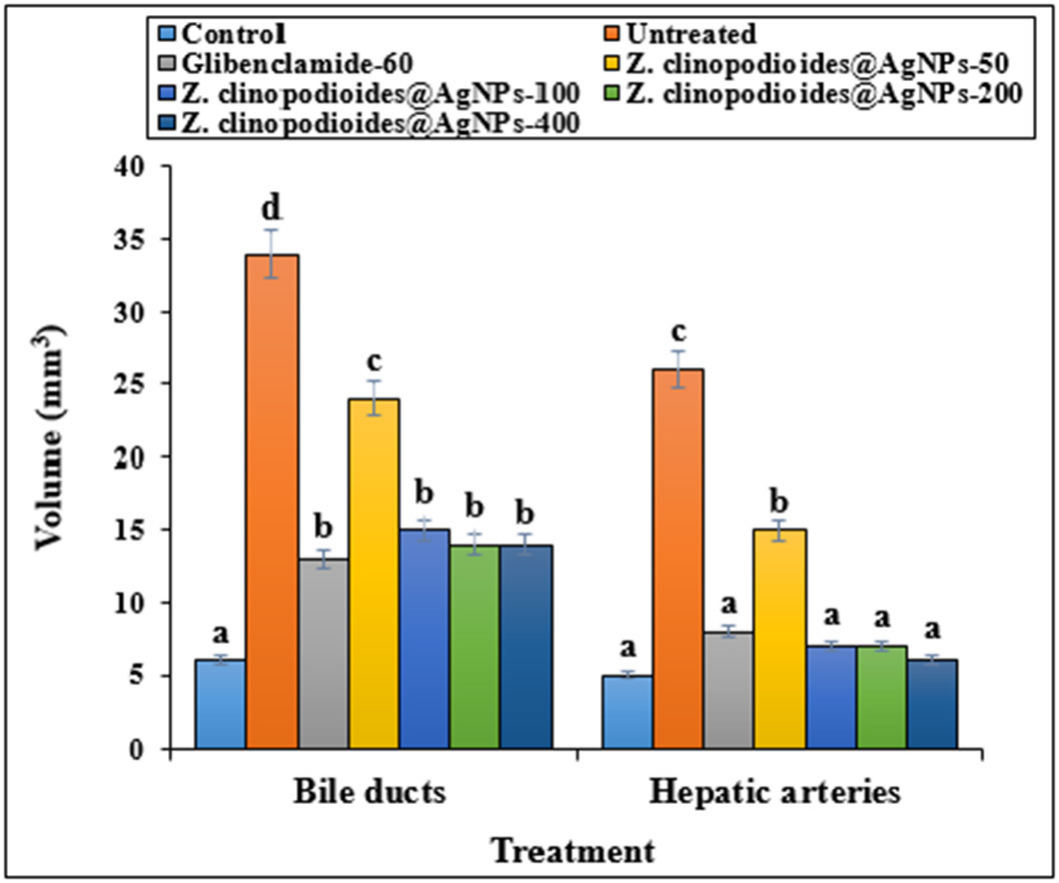
Volume of bile ducts and hepatic arteries in several groups. C: Control, UD: Untreated diabetic, G: Glibenclamide. The non‐similar letters indicate the significant difference between several groups (*p* ≤ .05).

**FIGURE 10:**
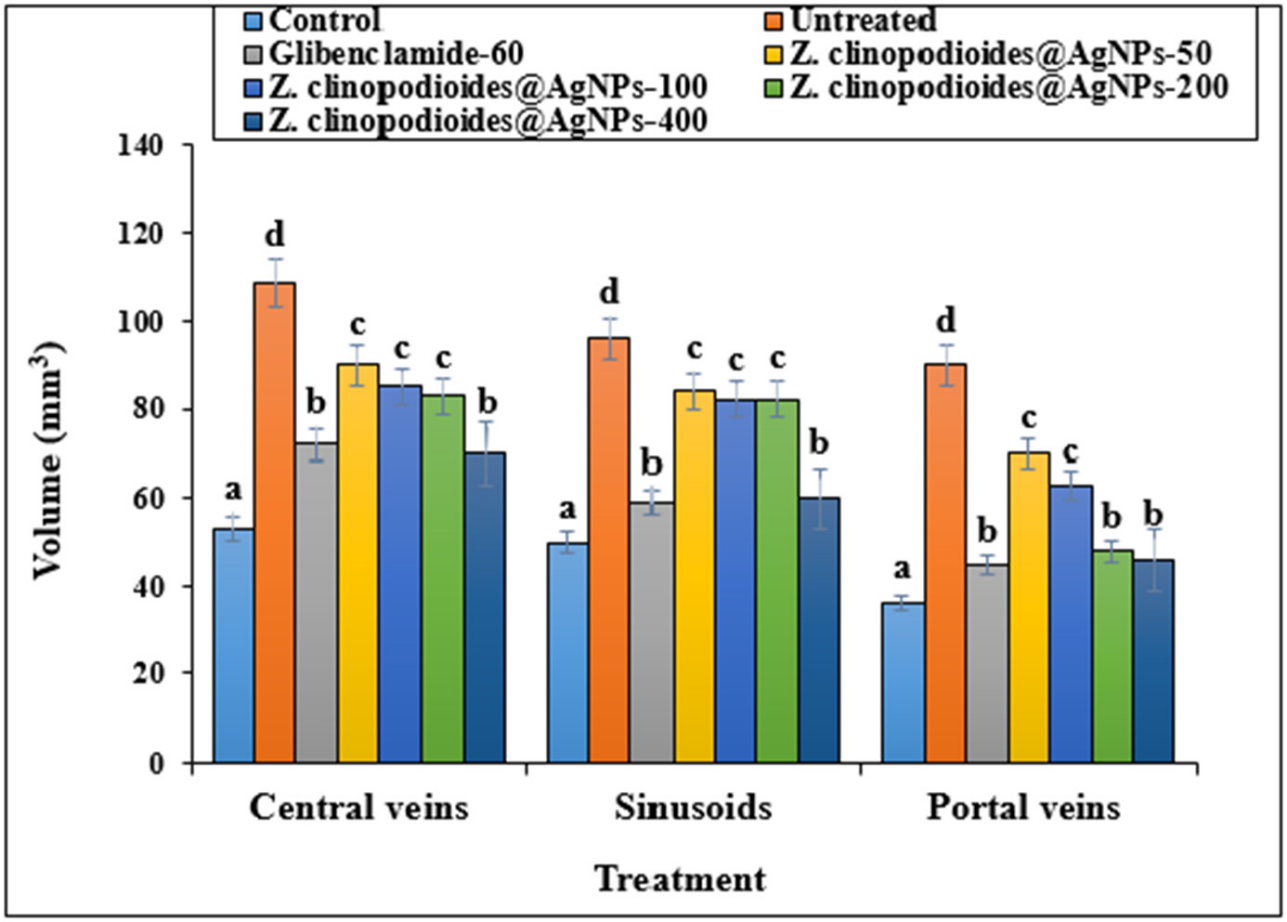
Volume of central veins, sinusoids, and portal veins in several groups. The non‐similar letters indicate the significant difference between several groups (*p* ≤ .05).

**FIGURE 11:**
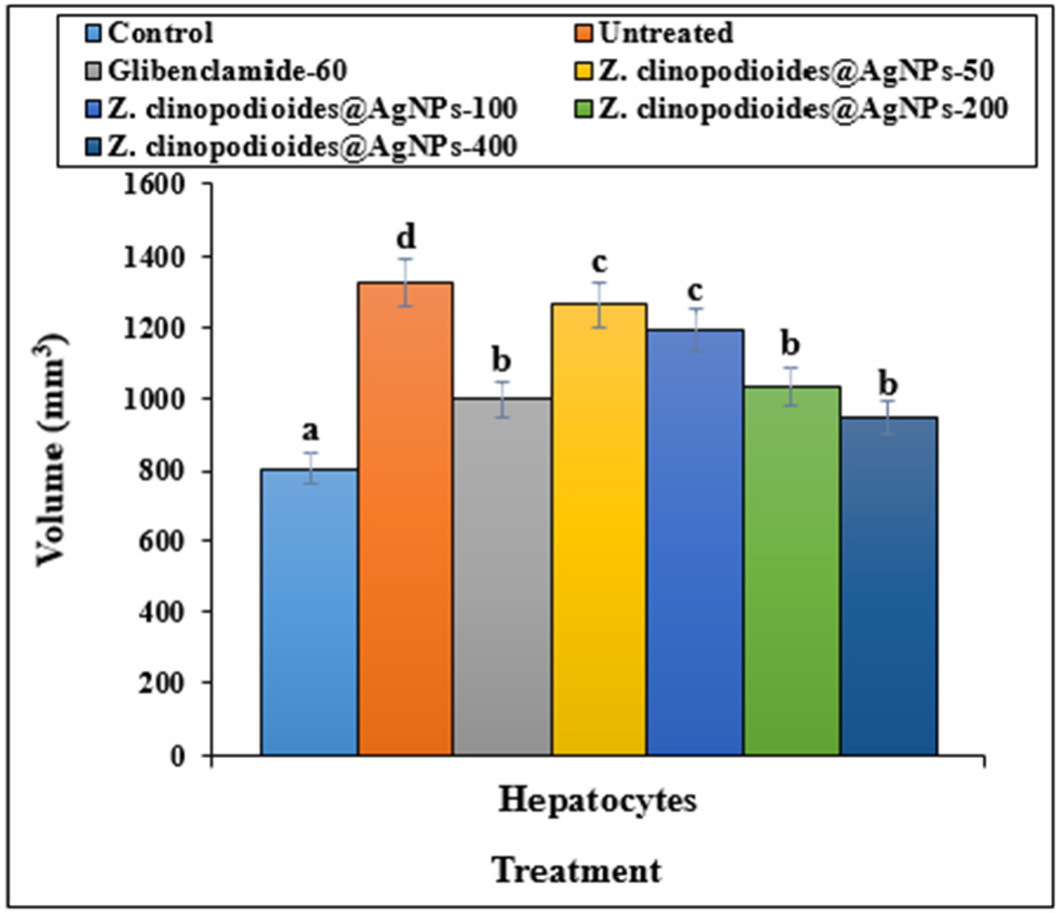
Volume of hepatocytes in several groups. The non‐similar letters indicate the significant difference between several groups (*p* ≤ .05).

In comparison with control models, the diabetic rats had significantly increased volumes of bile ducts, hepatic arteries, portal veins, central veins, sinusoids, and hepatocytes (*p* ≤ .05) (Figures 9, 10, 11). However, administration of AgNPs at all doses (especially AgNPs‐200 and AgNPs‐400) decreased the bile duct volumes, hepatic arteries, portal veins, central veins, sinusoids, and hepatocytes in comparison with the control group significantly (*p* ≤ .05).

**FIGURE 12:**
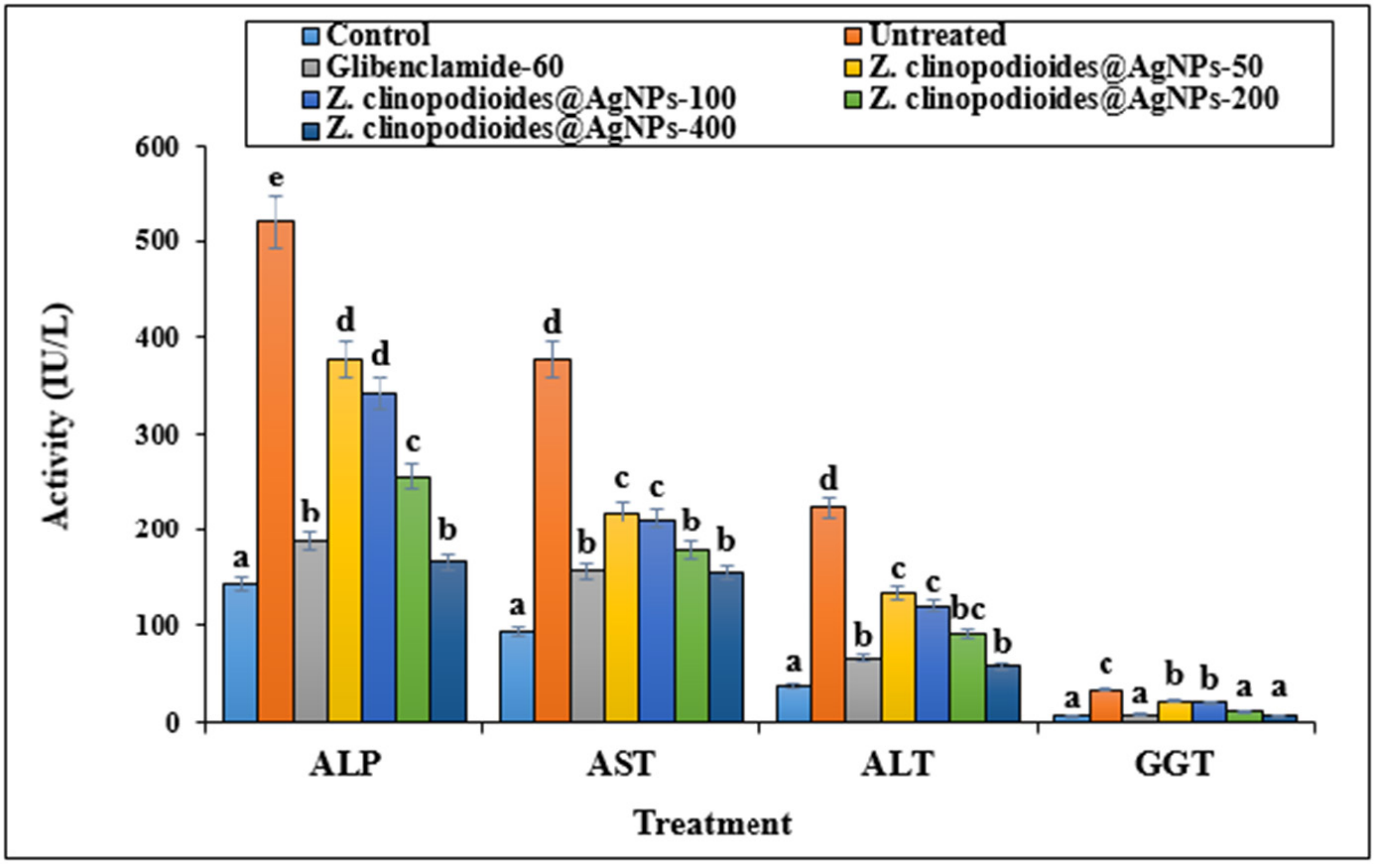
ALP, AST, ALT, and GGT levels in several groups. The non‐similar letters indicate the significant difference between several groups (*p* ≤ .05).

Figure 12 shows the estimated liver biochemical parameters, indicating that STZ‐induced toxicity augmented GGT, ALT, AST, and ALP significantly (*p ≤* .05) compared with the control group. Some doses of AgNPs decreased the augmented levels of GGT, ALT, AST, and ALP significantly (*p ≤* .05) compared with the control group.

## 4 CONCLUSION

We investigated an eco‐friendly approach for in situ immobilization of silver nanoparticles using an extract from *Ziziphora clinopodioides*. Unlike conventional methods, this approach does not involve the use of any toxic reducing and capping materials. The structure, morphology, and physicochemical characteristics of the nanoparticles were thoroughly determined. The findings of this research highlight the impact of combining modern scientific knowledge with past experiences, presenting new challenges and opportunities in diverse scientific fields, including medicine and pharmacy. Based on the results obtained, the biological nanoparticles synthesized using the plant extract played a critical role in controlling the levels of blood sugar in diabetic rats, while no adverse effects were observed or reported on the liver and pancreas tissues. These results signify that further research, coupled with the determination of the optimal concentration of biological nanoparticles, can pave the way for substantial advancements in controlling and treating different diseases.

## AUTHOR CONTRIBUTIONS

**Elham Pirabbasi:** Conceptualization (lead); investigation (lead); project administration (lead); writing – original draft (equal). **Mohammad Mahdi Zangeneh:** Data curation (equal); formal analysis (equal); investigation (equal); methodology (equal); writing – original draft (equal); writing – review and editing (equal). **Akram Zangeneh:** Investigation (equal); methodology (equal); software (equal); supervision (equal); writing – review and editing (equal). **Rohallah Moradi:** Investigation (equal); methodology (equal); software (equal); validation (equal). **Mojtaba Kalantar:** Project administration (equal).

## CONFLICT OF INTEREST STATEMENT

The present study authors state that they do not have any conflict of interests.

## ETHICS STATEMENT

This study does not involve any human tests, and all animal examinations were performed followed by receiving the ethical code “IR.SHOUSHTAR.REC.1399.010” from the Ethics Committee, Shoushtar Faculty of Medical Sciences.

## LIMITATION

Similar to other scientific studies, these research findings were also affected by some limitations:

1. To determine common symptoms of pathology, the clinical examinations were not performed every day or several times per day, but they were conducted periodically.
2. A very low volume of pancreas samples was collected from each rat, making the investigation of other immunological and biochemical parameters impossible.
3. The volume of pancreas tissue samples extracted from the rats was low; so, we could not investigate the expression of involved genes in them.

## Data Availability

The data that support the findings of this study are available from the corresponding author upon reasonable request.
